# Supplementary material for: BNT162b2-boosted immune responses six months after heterologous or homologous ChAdOx1nCoV-19/BNT162b2 vaccination against COVID-19
Source: Nat Commun. 2022 Aug 18;13:4872. doi: 10.1038/s41467-022-32527-2 (PMC9387891; doi:10.1038/s41467-022-32527-2)
Supplement: Supplementary file 1 — Supplementary Information [file 41467_2022_32527_MOESM1_ESM.pdf]

Supplementary Information:

BNT162b2-boosted immune responses six months after heterologous or homologous ChAdOx1nCoV-19/BNT162b2 vaccination against COVID-19

Georg M. N. Behrens, Joana Barros-Martins, Anne Cossmann, Gema Morillas Ramos, Metodi V. Stankov, Ivan Odak, Alexandra Dopfer-Jablonka, Laura Hetzel, Miriam Köhler, Gwendolyn Patzer, Christoph Binz, Christiane Ritter, Michaela Friedrichsen, Christian Schultze-Florey, Inga Ravens, Stefanie Willenzon, Anja Bubke, Jasmin Ristenpart, Anika Janssen, George Ssebyatika, Verena Krähling, Günter Bernhardt, Markus Hoffmann, Stefan Pöhlmann, Thomas Krey, Berislav Bošnjak, Swantje I. Hammerschmidt, Reinhold Förster

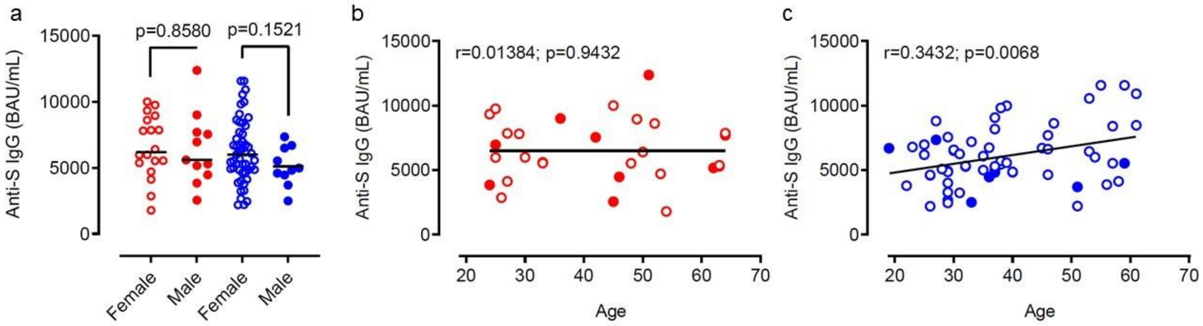

Suppl. Fig. 1: Correlation of age and sex to anti-S IgG after the third vaccination. a, Comparison of anti-S IgG after the third vaccination of females (open circles, n = 18 red, n = 51 blue) and males (filled circles, n =11 red, n = 10 blue) after homologous (red) or heterologous (blue) vaccination (two tailed Student's T test). b, Correlation of anti-S IgG and age by linear regression after homologous or c, heterologous vaccination labeled as in a. Source data are provided as a Source Data file.

a

Antigen	Conjugate	Clone	Order no.	Company	Dilution
CD14	BB700	MP9	566465	BD	1:100
CD16	BUV496	3G8	612944	BD	1:100
CD19	PECy7	HIB19	982410	BioLegend	1:200
CD20	BV421	2H7	302330	BioLegend	1:100
CD27	BUV805	L128	748704	BD	1:100
CD38	PerCP-eF710	HB7	46-0388-42	Invitrogen	1:100
IgD	BV480	IA6-2	566138	BD	1:200
IgM	AF647	MHM-88	314436	BioLegend	1:100
Viability	Zombie NIR™	-	423106	BioLegend	1:400
Anti-S BCR	mNeonGreen	Produced by T.Krey			5 µL / sample

b

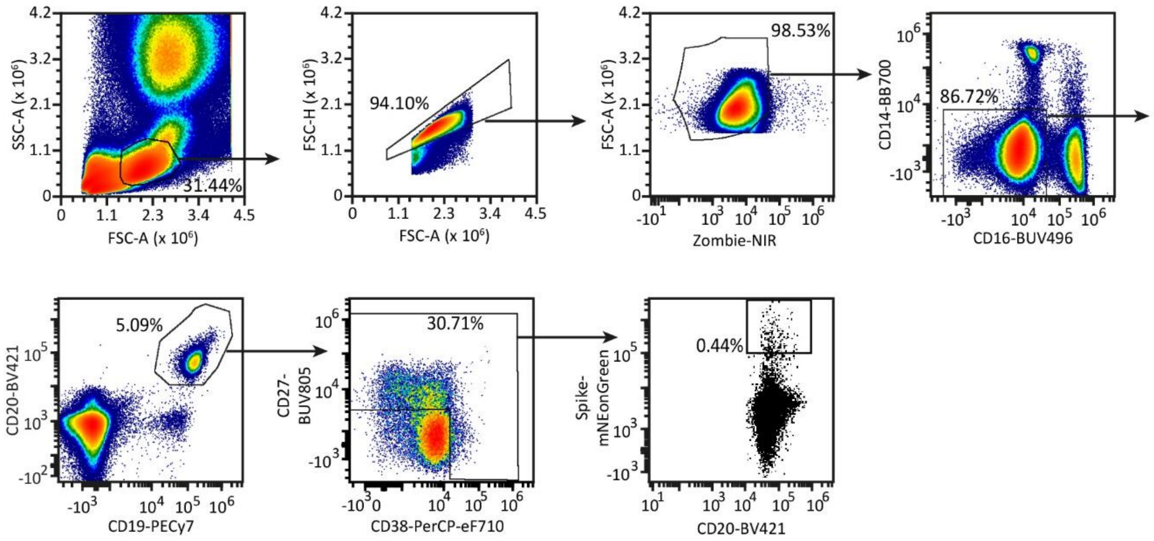

Suppl. Fig. 2 | Antibodies and gating strategy for B cells. a, antibody panel and b, gating strategy for SARS-CoV-2-S (Spike)-specific B cell populations in blood (see Fig. 1d). Pseudocolor plots show representative data from a female donor 283 days after priming with ChAd; 213 days after a second dose with BNT and 14 days after a third dose with BNT.

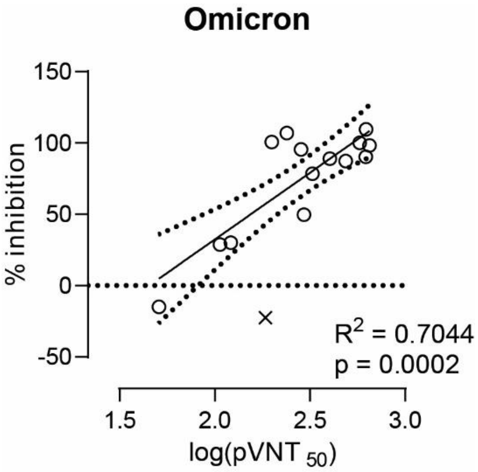

Suppl. Fig. 3 | Antibody neutralization measurements against the Omicron SARS-CoV-2 variant is positively correlated between the virus neutralization tests (sVNT) and pseudotyped virus neutralization tests (pVNT). Correlation (solid line) and 95% confidence intervals (dotted lines) between sVNT1:20 and antibody titers resulting in 50% reduction of luciferase activity in pVNT, indicated as pVNT50. Open circles, values from individual donors, outliers are marked with X and were defined as values with absolute residual value > 2 SD of all residual values in each group of samples. Correlation was calculated using single linear regression. Source data are provided as a Source Data file.

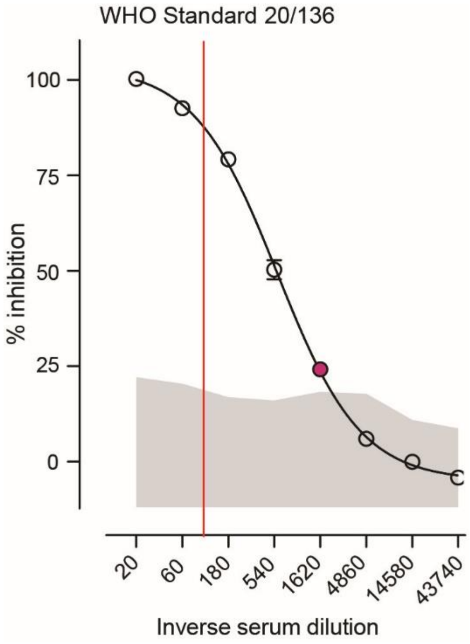

Suppl. Fig. 4 | Validation of sVNT: Precision calculation and titration of the First WHO International Standard for anti-SARS-CoV-2 Immunoglobulin. Titration of the First WHO International Standard for anti-SARS-CoV-2 Immunoglobulin (NIBSC code 20/136) in the Wuhan sVNT. The lower limit of detection is defined by the background signal of a pre-pandemic plasma pool (mean + 2SD; shaded area). Neutralizing sVNT titers were determined as the dilution with binding reduction > mean + 2SD of values from pre-pandemic plasma pool. Accordingly, the WHO International Standard 20/136 showed a titer of 1:1620 in the Wuhan sVNT (pink dot). By interpolating, the percent inhibition of the WHO Standard at a 1:100 serum dilution (red line) was determined as 86.9%. Source data are provided as a Source Data file.

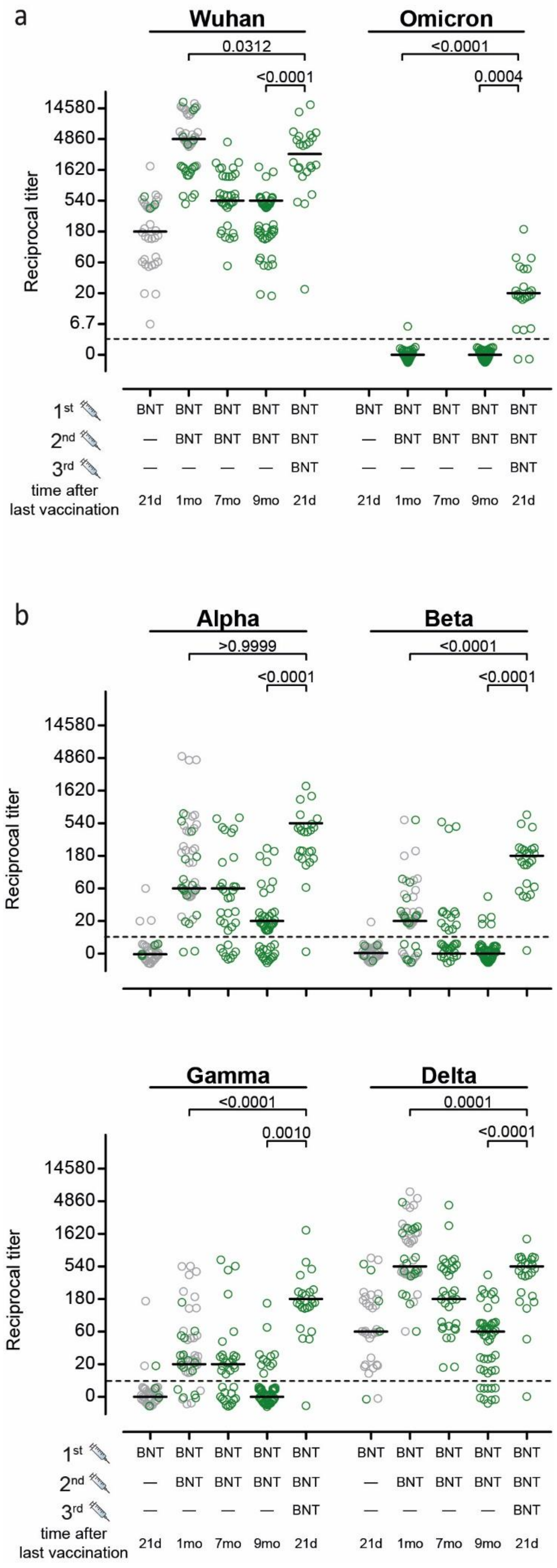

Suppl. Fig. 5 | Humoral immune response against all SARS-CoV-2 variants following homologous BNT162b2 (BNT) / BNT /BNT vaccination. Reciprocal titers of neutralizing antibodies against a, Wuhan and B.1.1.529 (Omicron) or b, B.1.1.7 (Alpha), B.1.351 (Beta), P.1 (B.1.1.28.1; Gamma), B.1.617.2 (Delta) SARS-CoV-2-S variants measured using the sVNT. Mixed effect analysis followed by Sidak's multiple comparison test (within groups). For better visualization of identical titer values, data were randomly and proportionally adjusted closely around the precise titer results. The dotted line represents the lower limit of detection. The symbols depicted in grey had been published before (15,19). Source data are provided as a Source Data file.

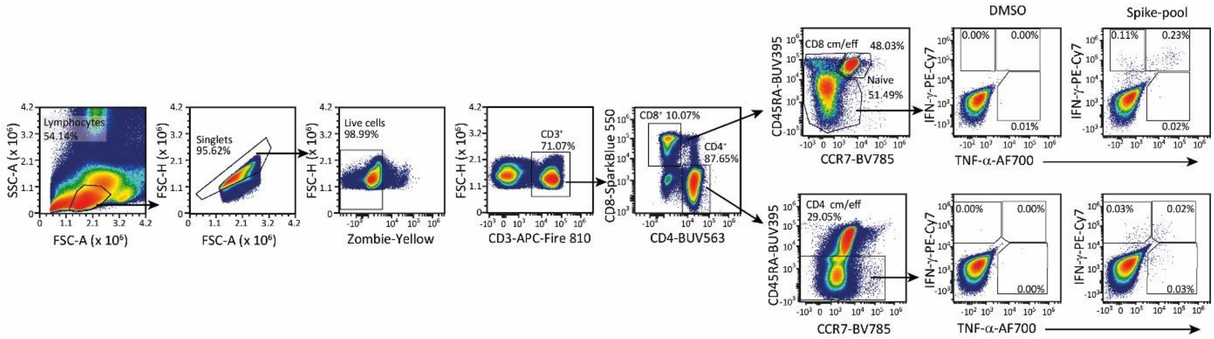

Suppl. Fig. 6 | Gating strategy used for detection of cytokine producing CD4+ and CD8+ T cells after *ex vivo* re-stimulation with DMSO or the pool of Spike-specific peptides for 12–16 hr as depicted in Fig. 3 a and b and Suppl. Fig. 7.

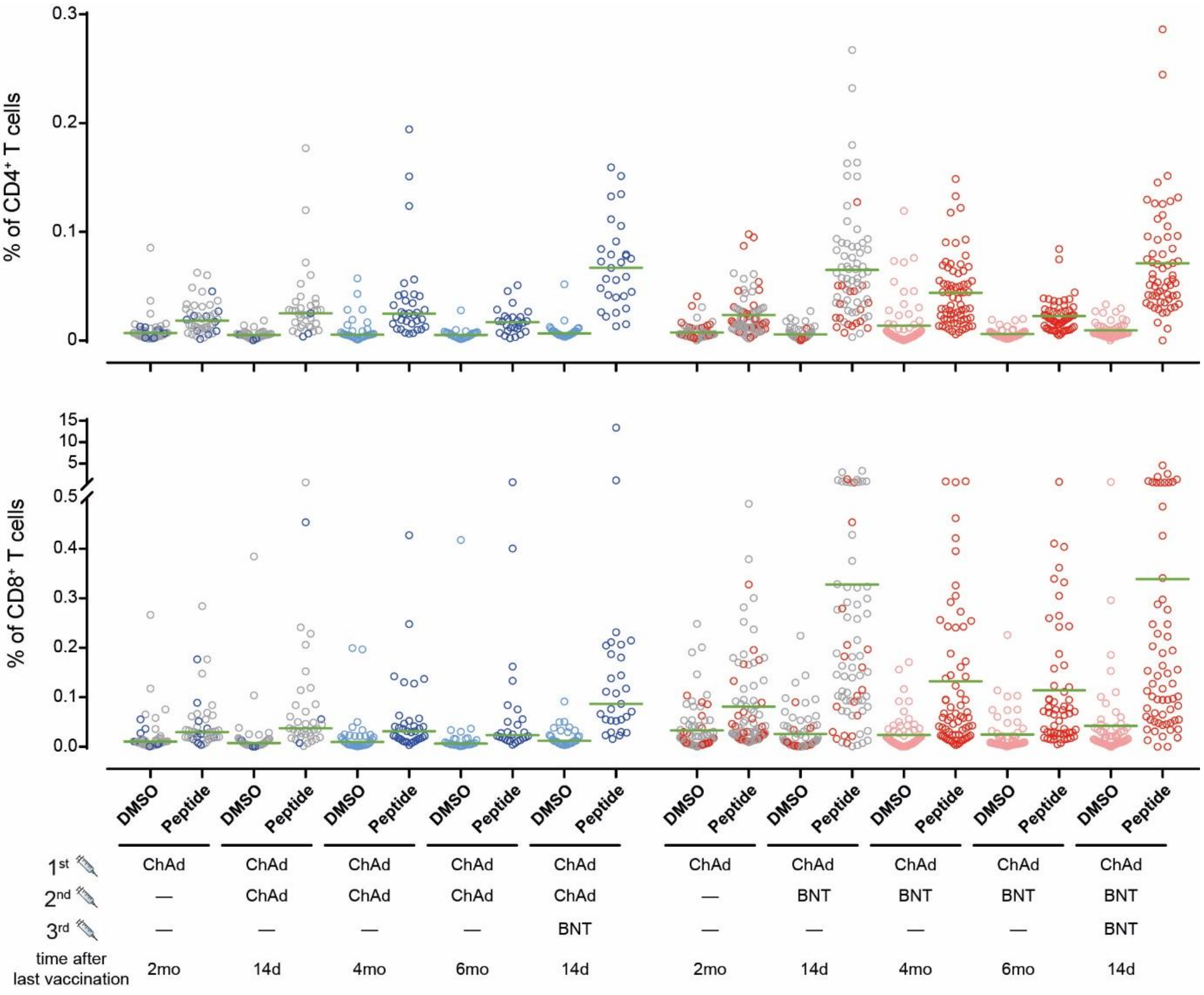

Suppl. Fig. 7 | Frequency of cytokine-producing CD4⁺ T cells and CD8⁺ T cells after *ex vivo* re-stimulation with DMSO or the pool of Spike-specific peptides for 12–16 hr. The dotted line represents the lower limit of detection. The symbols depicted in grey had been published before (15,19). Source data are provided as a Source Data file.

Type of variant-specific sVNT	Dilution of pre-pandemic plasma samples	Specificity (n=40)
Wuhan	1:6.7	100 %
Wuhan	1:20	100 %
Wuhan	1:60	100 %
Wuhan	1:180	100 %
Omicron	1:6.7	100 %
Omicron	1:20	100 %
Omicron	1:60	100 %
Omicron	1:180	100 %

Suppl. Table 1 | Specificity of the surrogate virus neutralization test (sVNT) as assessed by testing 40 different negative pre-pandemic plasma samples in different dilutions for neutralizing antibodies against the Wuhan and the Omicron variant.

Suppl. Note 1 | Validation of the sVNT

First, we characterized the precision, which describes the closeness of repeated individual measures of the analyte. Precision is expressed as the coefficient of variation defined as the ratio of standard deviation/mean (%). In detail, we analyzed seventeen different plasma samples covering a wide range of neutralizing titers. These samples were tested in three independent sVNT runs with six replicates of each sample. The three independent sVNT runs were performed on three different days. From these experiments, we calculated the percent inhibition for each replicate. Based on the results of the six replicates in a single run, we calculated the within-run coefficient of variation (CV). Further, we determined the standard deviation and the means from the three independent sVNT runs to calculate the between-run CV. Finally, to evaluate the CVs, we distinguished between data points close to the cut-off (i.e. lower limit of quantification data points) and all other data points (i.e. high and medium inhibition data points). 95.7% (n = 67 of 70) of the within-run CVs of the high and medium inhibition data points did not exceed 15%, while 81.0% (n = 34 of 42) of the within-run CVs of the lower limit of quantification data points did not exceed 20%. The remaining within-run CVs were in the range of between 16.3% and 20.8% (high and medium inhibition data points) and between 20.4% and 34.5% (lower limit of quantification data points). Similarly, 100.0% (n = 32/32) of the between-run CVs of the high and medium inhibition data points did not exceed 15%, while 83.3% (n = 10/12) of the between-run CVs of the lower limit of quantification data points did not exceed 20%. The remaining between-run CVs were in the range of between 20.3% and 31.5% (lower limit of quantification data points). Thus, the large majority of sVNT measures corresponded to the recommendations of the EMA guideline for bioanalytical method validation (EMEA/CHMP/EWP/192217/2009 Rev. 1 Corr. 2 accessed via https://www.ema.europa.eu/en/bioanalytical-method-validation on April 28, 2022) that applies for the highly regulated areas of animal toxicokinetic studies and all phases of clinical trials.

Next, we assessed the First WHO International Standard for anti-SARS-CoV-2 Immunoglobulin (NIBSC code 20/136) in the Wuhan sVNT. In general, the lower limit of detection is defined by the background signal of a pre-pandemic plasma pool (mean + 2SD, graphically displayed as shaded area in Suppl. Fig. 4). We defined the sVNT titer of a specific sample as the highest dilution that shows percent inhibition above the cut-off. The WHO International Standard 20/136 showed a titer of 1:1620 in the Wuhan sVNT (Suppl. Fig. 4, pink dot). The WHO arbitrarily assigned a value of 1000 neutralizing international units/mL (IU/mL) to the International Standard 20/136 (WHO reference number WHO/BS/2020.2402). Thus, the lower limit of detection is 1000 neutralizing IU/mL divided by 1620 = 0.62 neutralizing IU/mL.

Further, by interpolating, we determined the percent inhibition of the First WHO International Standard at a 1:100 serum dilution as 86.9% (Suppl. Fig. 4, red line). This value is close to the percent inhibition of 96.1% and 91.7% at a 1:100 reported for this WHO reference serum applying a commercial sVNT (Genscript) as reported in “Establishment of the WHO International Standard and Reference Panel for anti-SARS-CoV-2 antibody” (WHO reference number WHO/BS/2020.2402). Since the First WHO International Standard for anti-SARS-CoV-2 Immunoglobulin (NIBSC code 20/136) is no longer available, we could perform these tests only for the Wuhan sVNT but not for other VoC sVNTs. Finally, we determined the specificity of the sVNT by assessing 40 different pre-pandemic plasma samples. None of them showed any inhibition signal when tested in a 1:6.7, 1:20, 1:60 or 1:180 dilution for inhibition against the Wuhan and Omicron variant (Suppl. Table 1).
