# Supplementary material for: BNT162b2-boosted immune responses six months after heterologous or homologous ChAdOx1nCoV-19/BNT162b2 vaccination against COVID-19
Source: Nat Commun. 2022 Aug 18;13:4872. doi: 10.1038/s41467-022-32527-2 (PMC9387891; doi:10.1038/s41467-022-32527-2)
Supplement: Supplementary file 3 — Reporting Summary [file 41467_2022_32527_MOESM3_ESM.pdf]

nature research

Corresponding author(s): Georg Behrens
Reinhold Förster

Last updated by author(s): Jul 8, 2022

Reporting Summary

Nature Research wishes to improve the reproducibility of the work that we publish. This form provides structure for consistency and transparency in reporting. For further information on Nature Research policies, see our Editorial Policies and the Editorial Policy Checklist.

Statistics

For all statistical analyses, confirm that the following items are present in the figure legend, table legend, main text, or Methods section.

n/a	Confirmed	
☐	☒	The exact sample size (n) for each experimental group/condition, given as a discrete number and unit of measurement
☐	☒	A statement on whether measurements were taken from distinct samples or whether the same sample was measured repeatedly
☐	☒	The statistical test(s) used AND whether they are one- or two-sided<br>*Only common tests should be described solely by name; describe more complex techniques in the Methods section.*
☒	☐	A description of all covariates tested
☐	☒	A description of any assumptions or corrections, such as tests of normality and adjustment for multiple comparisons
☐	☒	A full description of the statistical parameters including central tendency (e.g. means) or other basic estimates (e.g. regression coefficient) AND variation (e.g. standard deviation) or associated estimates of uncertainty (e.g. confidence intervals)
☐	☒	For null hypothesis testing, the test statistic (e.g. F, t, r) with confidence intervals, effect sizes, degrees of freedom and P value noted<br>*Give P values as exact values whenever suitable.*
☒	☐	For Bayesian analysis, information on the choice of priors and Markov chain Monte Carlo settings
☒	☐	For hierarchical and complex designs, identification of the appropriate level for tests and full reporting of outcomes
☒	☐	Estimates of effect sizes (e.g. Cohen's d, Pearson's r), indicating how they were calculated

Our web collection on statistics for biologists contains articles on many of the points above.

Software and code

Policy information about availability of computer code

Data collection	Data were collected into Microsoft Excel 2016 or by Cytek Aurora spectral flow cytometer (Cytek) and SpectroFlo v2.2.0 (Cytek), SpectraMax iD3 microplate reader and SoftMax Pro v7.03 (Molecular devices), Hidex Sense Plate Reader and Hidex Sense Microplate Reader Software version 0.5.41.0, AESKU.READER and SoftMAX Pro v7.03. No custom algorithms or software.
Data analysis	Graphpad Prism V8.4 or V9, SPSS 20.0.0, Gen5 2.01 Software, SpectroFlo (Cytek), FCS Express V7 (De Novo Software), Hidex Sense Microplate Reader Software (version 0.5.41.0), SoftMAX Pro v7.03.

For manuscripts utilizing custom algorithms or software that are central to the research but not yet described in published literature, software must be made available to editors and reviewers. We strongly encourage code deposition in a community repository (e.g. GitHub). See the Nature Research guidelines for submitting code & software for further information.

Data

Policy information about availability of data

All manuscripts must include a data availability statement. This statement should provide the following information, where applicable:

- Accession codes, unique identifiers, or web links for publicly available datasets
- A list of figures that have associated raw data
- A description of any restrictions on data availability

All requests for raw and analyzed data that underlie the results reported in this article will be reviewed by the CoCo Study Team, Hannover Medical School (cocostudie@mh-hannover.de) to determine whether the request is subject to confidentiality and data protection obligations. Data that can be shared will be released via a material transfer agreement.

Field-specific reporting

Please select the one below that is the best fit for your research. If you are not sure, read the appropriate sections before making your selection.

☒ Life sciences ☐ Behavioural & social sciences ☐ Ecological, evolutionary & environmental sciences

For a reference copy of the document with all sections, see nature.com/documents/nr-reporting-summary-flat.pdf

Life sciences study design

All studies must disclose on these points even when the disclosure is negative.

Sample size

Sample size calulations were explained previously (see Reporting Summary Barros-Martin J et al. Nat Med 2021). We estimated that a sample size of 30 subjects in each arm is sufficient to detect a clinically meaningful difference within each group assuming that Spike protein specific IgG doubles from first vaccination (mean 95 RU/mL with a standard deviation of 113 RU/mL) using a two-tailed paired t-test of differences between means with a 95% power and a 1% level of significance. As an additional control group, n=57 BNT/BNT prime/boost vaccines were followed up similarly. The power calculation was performed with G*Power, Version 3.1.9.6.

Data exclusions

One participant with serologic evidence for SARS-CoV-2 infection before vaccination was excluded and three individuals with novel anti-NCP antibodies (two in the ChAd/ChAd and one in the BNT/BNT group) suggesting seroconversion during the observation period after the second vaccination were censored and not further analyzed.

Replication

Acquisition of FACS data for phenotyping and intracellular cytokine staining were done as state-of-the-art single sample measurements. We were unable to run samples for FACS analysis in duplicate because the number of cells isolated from the given volume of blood is limited. However, we verified the results of intracellular cytokine staining by including negative controls for stimulation. sVNT measurements were done in duplicate samples. By duplicate measurements, we could verify the reproducibility of the sVNT findings.

Randomization

Randomization was not performed, since ChAd boosting after ChAd priming was not recommended for persons less than 60 years of age by the German Standing Committee for Vaccination (STIKO) at that time. However, the STIKO recommendation allowed ChAd boosting for that age group when requested by the vaccinee. Thus, all vaccinees of the study were unrestricted in their choice between ChAd or BNT for booster vaccination. STIKO recommended mRNA booster vaccination for health care professionals (and others) on Nov. 18, 2021 and vaccinees in this study received BNT almost exclusively through the vaccination campaing at Hannover Medical School.

Blinding

Investigators performing serology or FACS analyses were blinded to participant's details and vaccination schedule.

Reporting for specific materials, systems and methods

We require information from authors about some types of materials, experimental systems and methods used in many studies. Here, indicate whether each material, system or method listed is relevant to your study. If you are not sure if a list item applies to your research, read the appropriate section before selecting a response.

Materials & experimental systems

n/a	Involved in the study	
☐	☒	Antibodies
☐	☒	Eukaryotic cell lines
☒	☐	Palaeontology and archaeology
☒	☐	Animals and other organisms
☐	☒	Human research participants
☐	☒	Clinical data
☒	☐	Dual use research of concern

Methods

n/a	Involved in the study	
☒	☐	ChIP-seq
☐	☒	Flow cytometry
☒	☐	MRI-based neuroimaging

Antibodies

Antibodies used

Antigen, Conjugate, Company, Order no., Clone, Lot, Dilution
CD14, BB700, BD, 566465, MφP9, 0267076, 1:100
CD16, BUV496, BD, 612944, 3G8, 0288806, 1:100
CD19, PECy7, Biolegend, 982410, HIB19, B317196, 1:200
CD20, BV421, Biolegend, 302330, 2H7, B313870, 1:100
CD27, BUV805, BD, 748704, L128, 1091292, 1:100
CD38, PerCP-eF710, Invitrogen, 46-0388-42, HB7, 2290343, 1:100
IgD, BV480, BD, 566138, IA6-2, 1117442, 1:200
IgM, Af647, Biolegend, 314536, MHM-88, B288547, 1:100
Viability, Zombie NIR™, Biolegend, 423106, -, B323372, 1:400
Anti-S BCR, mNeonGreen, Produced by T. Krey, 5µg/sample
CD3, APC-Fire 810, BioLegend, 344858, SK7, B331674, 1:50

	CD4, BUV563, BD, 741353, RPA-T4, 0295029, 1:200<br>CD8, SparkBlue 550, Biolegend, 344760, SK1, B326454, 1:200<br>CD45RA, BUV395, BD, 740298, HI100, 0295008 and 1270969, 1:200<br>CCR7, BV785, Biolegend, 353230, G043H7, B335328, 1:50<br>TNFa, AF700, Biolegend, 502928, MAb11, B337546, 1:50<br>IFNg, Pe-Cy7, BioLegend, 506518, B27, B326674, 1:100<br>Viability, Zombie Yellow™, BioLegend, 423104, -, B272131, 1:400<br>Anti-His coupled to HRP, homemade, clone 3D5, 1:3000
Validation	All commercially available antibodies used in this study have been validated for research purpose by the manufacturer. Validation data for each catalogue number is available on the manufacturer's website. All of BioLegend products undergo industry-leading rigorous quality control (QC) testing to ensure the highest level of performance and reproducible results. Each lot is compared to an internally established "gold standard" to maintain lot-to-lot consistency. BioLegend also conducts wide-scale stability studies to guarantee an accurate shelf-life for their products.<br>- Specificity testing of 1-3 target cell types with either single- or multi-color analysis (including positive and negative cell types).<br>- Once specificity is confirmed, each new lot must perform with similar intensity to the in-date reference lot. Brightness (MFI) is evaluated from both positive and negative populations.<br>- Each lot product is validated by QC testing with a series of titration dilutions.<br><br>Anti-S BCR has been produced by T. Krey (Institute of Biochemistry, University of Lübeck, 23562 Lübeck, Germany). Validation was made in house using healthy control samples collected before the first appearance of the SARS-CoV-2 (January 2020) and equally processed COVID-19 patient samples.

Eukaryotic cell lines

Policy information about cell lines

Cell line source(s)	293T, VERO
Authentication	Cell lines were originally obained from ATCC and were not authenticated further.
Mycoplasma contamination	Cell lines were not tested for mycoplasma contamination
Commonly misidentified lines<br>(See ICLAC register)	This study was not done using commonly misidentified cell lines.

Human research participants

Policy information about studies involving human research participants

Population characteristics	Age and sex are provided in Table 1
Recruitment	Participants for this analysis were from the COVID-19 Contact (CoCo) Study, which started in March 2020 and is an ongoing observational study monitoring anti-SARS-CoV-2 IgG immunoglobulin and immune responses in n=1,493 health care professionals (HCP) at Hannover Medical School. All study participants gave written informed consent. According to German regulations, HCP were prioritized for SARS-CoV2 vaccination and received first doses of the BNT from Jan 6th and ChAd from Feb 16th, 2021 onwards. Second vaccination of ChAd primed HCP started on May 3rd and individuals could freely choose to receive either ChAd or BNT for second vaccination. On a first come first served basis, 130 ChAd primed vaccinees of the CoCo Study cohort were invited to donate blood before the boosting of ChAd primed vaccinees started. Follow up continued until after the third vaccinations were recommended and performed. In addition, n=57 BNT/BNT vaccinated HCP were followed up until 3 weeks after third BNT vaccination. Recruitment was non-randomized and the vaccination not blinded. Thus potential bias between groups could have been introduced by the self-selection of participants. Initial decision on whether to receive a vector-based or mRNA-based vaccine could have been influenced by e.g. gender, age, educational level, health status, perceived risk for COVID-19 or other conditions. In addition, self-perceived risk for adverse drug reactions or previous adverse reactions after first vaccination could have led to potential bias.
Ethics oversight	Institutional Review Board of Hannover Medical School (8973_BO-K_2020).

Note that full information on the approval of the study protocol must also be provided in the manuscript.

Clinical data

Policy information about clinical studies

All manuscripts should comply with the ICMJE guidelines for publication of clinical research and a completed CONSORT checklist must be included with all submissions.

Clinical trial registration	N/A
Study protocol	N/A
Data collection	N/A
Outcomes	N/A

Flow Cytometry

Plots

Confirm that:

☒ The axis labels state the marker and fluorochrome used (e.g. CD4-FITC).

☒ The axis scales are clearly visible. Include numbers along axes only for bottom left plot of group (a 'group' is an analysis of identical markers).

☒ All plots are contour plots with outliers or pseudocolor plots.

☒ A numerical value for number of cells or percentage (with statistics) is provided.

Methodology

Sample preparation	Blood was collected by venipuncture and samples transported to the lab within 6 hours. We separated plasma from EDTA or lithium heparin blood (S-Monovette, Sarstedt) and stored it at minus 80°C until use. We used full blood or isolated PBMCs by Ficoll gradient centrifugation for flow cytometry analysis. Cell supernatants for cytokine analysis were frozen and stored at minus 80°C until use. All detailed procedures are described in Methods.
Instrument	Cytek Aurora (Cytek), LSR II flow cytometer (Becton Dickinson, Heidelberg, Germany), AESKU.READER (AESKU.GROUP, Wendelsheim, Germany), SpectraMax iD3 microplate reader (Molecular Devices), Hidex Sense Plate Reader
Software	SpectroFlo (Cytek), FCS Express (De Novo Software), BD FACSDiva v8.0.1 Software, Gen5 2.01 Software, Hidex Sense Microplate Reader Software (version 0.5.41.0), SoftMAX Pro v7.03
Cell population abundance	N/A - this study did not include sorting of the cells.
Gating strategy	Spike-specific B cells: FSC-A/SSC-A > Single cells using FSC-A/FSC-H > viable cells using FSC-A/Zombie > CD14neg/CD16neg > CD20pos/CD19pos > CD27pos/CD38pos > CD20 pos/anti-S BCR.<br>Spike-specific T cells: FSC-A/SSC-A > Single cells using FSC-A/FSC-H > viable cells using FSC-A/Zombie > FSC-H/CD3pos > CD4pos/CD8pos to discrimitate CD4 and CD8 T cells > each population CD45RApos/CCR7pos > IFN gamma/TNF alpha double/single pos cells. Gating strategies are shown in Suppl. Figure 2 and 6.

☒ Tick this box to confirm that a figure exemplifying the gating strategy is provided in the Supplementary Information.
